# Visualization of the epicardial conduction through the Marshall bundle using the LUMIPOINT™ software

**DOI:** 10.1002/joa3.12879

**Published:** 2023-05-31

**Authors:** Rintaro Hojo, Tomoyuki Arai, Takashi Kimura, Masao Takahashi, Seiji Fukamizu

**Affiliations:** ^1^ Department of Cardiology Tokyo Metropolitan Hiroo Hospital Tokyo Japan

**Keywords:** activation map, atrial tachycardia, catheter ablation, high‐resolution mapping, Marshall bundle

## Abstract

The LUMIPOINT™ software allows visualization of arrhythmia circuits through the MB. In cases where the full extent of the arrhythmia circuit cannot be identified and epicardial conduction is suspected, it is better to perform the analysis while adjusting the confidence slider in LUMIPOINT™.

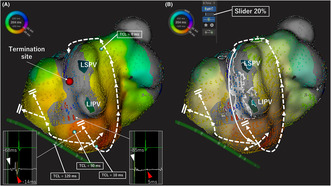

In a previous study,^1^, ^2^ we reported that the RHYTHMIA™ mapping system using an ORION™ catheter (Boston Scientific, Marlborough, MA) can record conduction through the Marshall bundle (MB) as a tiny potential from the endocardial side. Recording the course of the MB from the endocardial side is useful for determining the ablation sites. However, the timing of these tiny potentials is not reflected in the activation map because their timings are not consistent with those of their surroundings. LUMIPOINT™ (Boston Scientific) software visualizes the propagation of all deflections within a bounded window of interest, referred to as the LUMIPOINT™ activation window. Considering this new software would allow us to visualize conduction through the MB, we reanalyzed a previously reported case.

A 73‐year‐old woman with a history of mitral valve plasty, pulmonary vein isolation, and linear ablation of the roof and anterior wall of the left atrium (LA) was admitted for atrial tachycardia (AT) ablation. An electrophysiological study was performed after the patient provided informed consent. The activation map of LA using the RHYTHMIA™ mapping system revealed a centrifugal pattern at the lower lateral mitral isthmus (Figure 1A). The postpacing interval at the left posterior wall and the center of the centrifugal propagation site was equal to the tachy‐cycle length (TCL). Those at the mitral isthmus beneath the scar area and mitral annulus were longer than the TCL by 50 ms (Figure 1A). This indicated that the AT propagated around the left pulmonary vein in a counterclockwise manner and that the MB bypassed the scar area of the mitral isthmus. The conduction through the MB was recorded as a tiny potential from the endocardial side (Figure 1A, red arrow), which did not reflect the activation map. Although we considered inserting electrodes into the vein of Marshall, the coronary sinus venography could not detect the vein of Marshall. Finally, the AT was terminated in 20 seconds by 40 W radiofrequency application targeting the tiny potential of the MB recorded from the endocardial side (Figure 1A, red tag). This case was reanalyzed using the LUMIPOINT™ software. In general, LUMIPOINT™ is used with the confidence slider, which is a function that changes the tolerance for consistency with the surrounding points, set to 85%–100%. In this setting, the potential on the endocardial side of the MB was not highlighted (Supplementary Video S1 [left side]). By lowering the confidence slider value of the LUMIPOINT™ software from 100% to 20%, we were able to visualize the circuit on the epicardial side, which was missing from the activation map (Figure 1B & Supplementary Video S1 [right side]).

**FIGURE 1:**
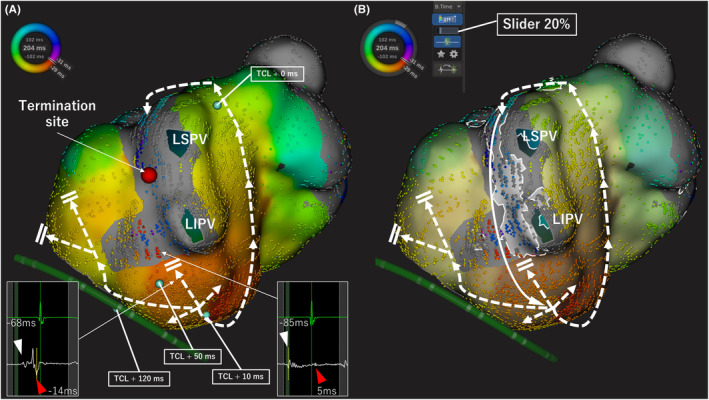
(A) Activation map of atrial tachycardia. Postpacing intervals were calculated using the blue tags. Postpacing was similar to the tachycardia cycle length at the centrifugal activation site and the posterior wall. According to the activation map, the preceding potentials (white arrowheads) were suspected to be the far‐field potentials of the Marshall bundle, while the following potentials (red arrowheads) were endocardial potentials. The atrial tachycardia was terminated by catheter ablation targeting the tiny potential of the MB recorded from the endocardial side (red tag). (B) The activation map was reanalyzed using LUMIPOINT™ software. By lowering the confidence slider value of the LUMIPOINT™ software to 20%, we were able to visualize the circuit on the epicardial side, which was missing from the activation map. LIPV, left inferior pulmonary vein; LSPV, left superior pulmonary vein; TCL, tachy‐cycle length.

By lowering the confidence mask level to 0.01 mV, that is displaying the area below 0.01 mV as a scar area, activation of MB in the low voltage region can be visualized. (Figure 2A, confidence mask <0.01 mV). However, the activation of MB near the centrifugal pattern can only be visualized with LUMIPOINT™ (Figure 2A,B dotted line circle).

**FIGURE 2:**
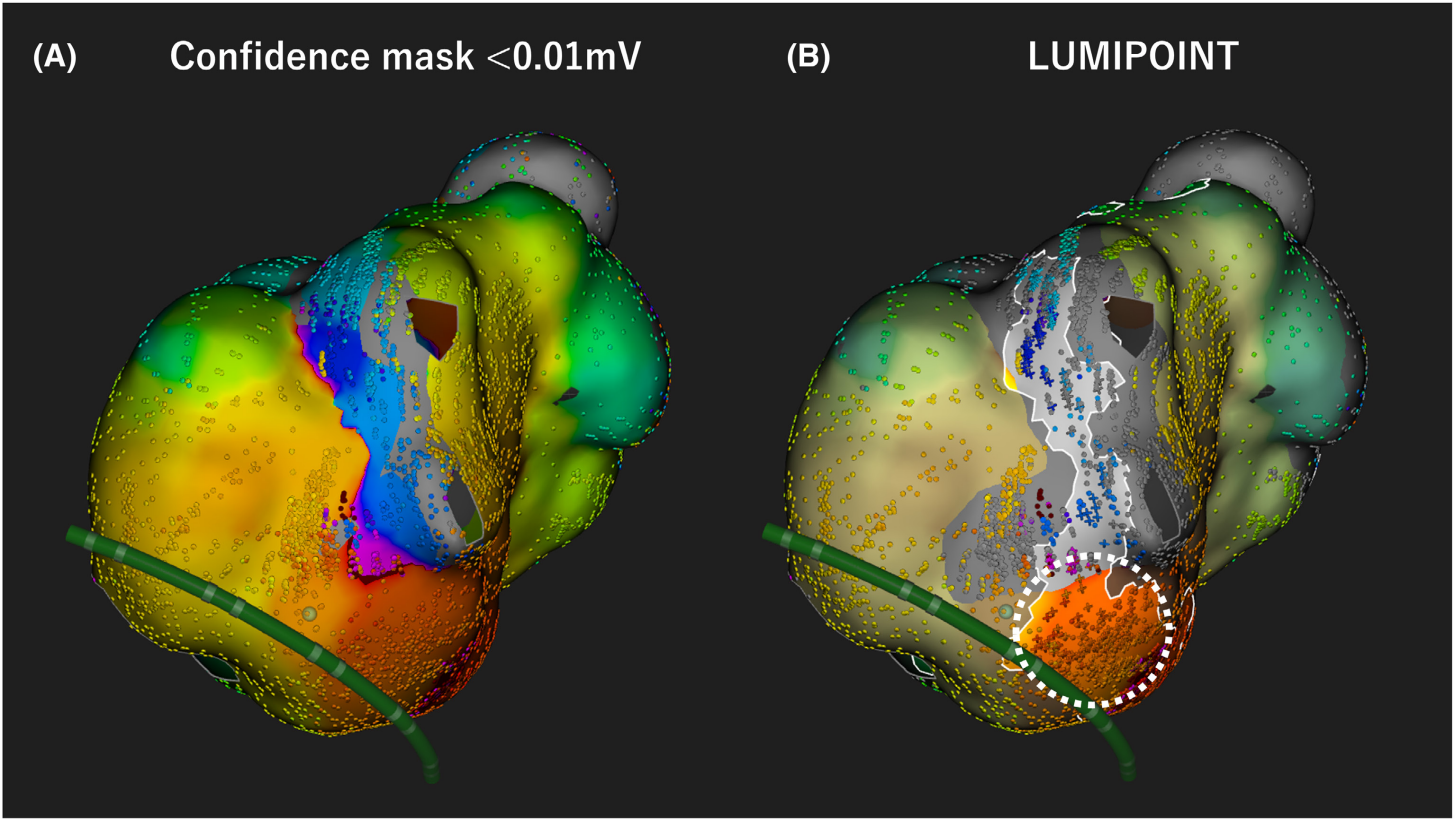
(A) By lowering the Confidence mask level to 0.01 mV, activation of Marshall bundle in the low voltage region can be visualized. (B) The activation of Marshall bundle near the centrifugal pattern (dotted circle line) can be visualized with LUMIPOINT™.

The most important aspect of this case was that the epicardial conduction through the MB was observed in the scar area. In cases with the Marshall vein, where insertion of a catheter is not suitable, recording its potential and confirming the MB course are important in determining the site of catheter ablation. The ORION™ catheter has a narrow interelectrode distance, theoretically unsuitable for recording potentials at distant sites. The MB is in close proximity to the endocardial side and could have been recorded. The higher the confidence slider value, the more consistent the surrounding potential; therefore, the confidence slider value is usually set high. On the other hand, by lowering the confidence slider value, it is possible to visualize the unseen epicardial pathway, which is not consistent with the timing of surrounding deflections.

In the RHYTHMIA™ mapping system, it is possible to record the MB potential from the endocardial side. In addition, the LUMIPOINT™ software allows visualization of arrhythmia circuits through the MB. In cases where the full extent of the arrhythmia circuit cannot be identified and epicardial conduction is suspected, it is better to perform the analysis while adjusting the confidence slider in LUMIPOINT™.

## CONFLICT OF INTEREST STATEMENT

Authors declare no conflict of interests for this article.

## ETHICS STATEMENT

N/A.

## PATIENT CONSENT STATEMENT

An electrophysiological study was performed after the patient provided informed consent.

## CLINICAL TRIAL REGISTRATION

N/A.

## Supporting information


**Video S1** The propagation map using LUMIPOINT™ with 100% and 20% slider levels on the left and right sides, respectively.
